# MicroRNA regulates the toxicological mechanism of four mycotoxins in vivo and in vitro

**DOI:** 10.1186/s40104-021-00653-4

**Published:** 2022-02-24

**Authors:** Jia Chen, Shuhua Yang, Peng Li, Aibo Wu, Eugenie Nepovimova, Miao Long, Wenda Wu, Kamil Kuca

**Affiliations:** 1 College of Animal Science & Veterinary Medicine, Shenyang Agricultural University, Shenyang, 110866 China; 2 SIBS-UGENT-SJTU Joint Laboratory of Mycotoxin Research, CAS Key Laboratory of Nutrition, Metabolism and Food Safety, Shanghai Institute of Nutrition and Health, University of Chinese Academy of Sciences, Chinese Academy of Sciences, Shanghai, 200031 China; 3 Department of Chemistry, Faculty of Science, University of Hradec Kralove, Hradec Kralove, 50003 Czech Republic; 4 MOE Joint International Research Laboratory of Animal Health and Food Safety, College of Veterinary Medicine, Nanjing Agricultural University, Nanjing, 210095 China; 5 Biomedical Research Center, University Hospital Hradec Kralove, Hradec Kralove, 50003 Czech Republic

**Keywords:** Histone, Methylation, MicroRNA, Mycotoxin

## Abstract

Mycotoxins can cause body poisoning and induce carcinogenesis, often with a high mortality rate. Therefore, it is of great significance to seek new targets that indicate mycotoxin activity and to diagnose and intervene in mycotoxin-induced diseases in their early stages. MicroRNAs (miRNAs) are physiological regulators whose dysregulation is closely related to the development of diseases. They are thus important markers for the occurrence and development of diseases. In this review, consideration is given to the toxicological mechanisms associated with four major mycotoxins (ochratoxin A, aflatoxin B1, deoxynivalenol, and zearalenone). The roles that miRNAs play in these mechanisms and the interactions between them and their target genes are explained, and summarize the important role of histone modifications in their toxicity. As a result, the ways that miRNAs are regulated in the pathogenicity signaling pathways are revealed which highlights the roles played by miRNAs in preventing and controlling the harmful effects of the mycotoxins. It is hoped that this review will provide a theoretical basis for the prevention and control of the damage caused by these mycotoxins.

## Introduction

Mycotoxins are secondary metabolites produced by fungi that are widely found in feed and food. The most troublesome mycotoxins include: ochratoxin A (OTA), aflatoxin B1 (AFB1), deoxynivalenol (DON), and zearalenone (ZEA) [1]. Worldwide, about 60–80% of feed is contaminated with mycotoxins each year [2]. In 3507 feed samples from different regions of China, it was found that the contamination rates of AFB1, DON, and ZEA reached 81.9, 96.4, and 96.9% respectively, and more than 81.5% of feed ingredients and 95.7% of complete feeds were found to be contaminated by these mycotoxin combinations [3]. Filamentous fungi can grow on a variety of grains, causing mycotoxin production and growth when oxygen remains in silage, and fungi that are tolerant to carbon dioxide and organic acids can continue to grow [4]. Any feed that is contaminated with mycotoxins during the planting, harvesting, transportation, and storage, etc. stages can cause the poisoning of livestock and poultry [5]. Studies have shown that mycotoxins can exert toxic effects through a variety of signaling pathways (e.g. MAPK, NRF2, Wnt, P53, and PI3K), causing cytotoxicity, oxidative stress, and genotoxicity to the liver and kidneys. Mycotoxins also can significantly increase the risk of malformation, cancer, diabetes, and nephritis in humans [6].

MicroRNAs (miRNAs) are a class of endogenous non-coding small molecules with lengths of about 21–28 nt. They are commonly found in animals, plants, and viruses. MiRNAs are even involved in gene regulation in single-celled organisms, green algae, and *Chlamydomonas* species [7]. In-depth studies have been carried out on miRNAs in recent years. MiRNAs have been revealed to be involved in the regulation of many physiological diseases, influencing a wide range of processes, such as, apoptosis, differentiation, necrosis, and inflammation [8]. It has thus been found that the direct or indirect stimulation of some chemical substances or stimulants can cause the abnormal expression of miRNAs in the body, such as Selenium, Quercetin, Apigenin, Tanshinone IIA, Cinnamaldehyde [9–13]. At the same time, the mechanisms by which miRNAs act are of great significance to the diagnosis, treatment, and prognosis of diseases, as well as the study of genetic drugs and tumorigenesis.

Therefore, in addition to ensuring the processing and storage of feed, the prevention of mycotoxins can effectively intervene the occurrence of diseases by finding effective miRNAs and inhibiting the transcription and translation of pathogenic genes. The reasonable and effective use of miRNAs as a tool to evaluate the exposure of toxins in the body and regulate the expression of pathogenic target genes and enzymes is of great significance for the prevention and treatment of mycotoxin poisoning. In this paper, we summarize the toxicological mechanisms associated with the four major mycotoxins: OTA, AFB1, DON, and ZEA. The signaling pathways involved and the regulatory roles played by miRNAs are outlined. The aim is to provide a reference for predicting and therefore reducing the effects of mycotoxin exposure at the molecular level.

### Toxicological significance of miRNAs in mycotoxins

MiRNAs can be viewed as characteristic biomarkers whose levels can be directly measured in serum, urine, and saliva. Therefore, they are useful biological indicators and have broad prospects in disease detection and prevention. As they play key roles in regulating the transcription and expression of genes in eukaryotes, miRNAs have been reported to affect the expression of most genes in mammals, and each miRNA also has more than 300 highly conserved targets [14, 15]. Therefore, finding miRNAs to inhibit the transcription and translation of the target genes of diseases is an effective way of intervening in the progression of those diseases.

Clearly, identifying effective biological detoxification agents for mycotoxins is of great importance for the prevention and treatment of mycotoxin poisoning. However, the reasonable and effective use of miRNAs as a way of assessing endotoxin exposure and regulating pathogenic target genes and protein expression can be expected to be a powerful tool that can be used to help achieve the same aims. At present, it has been shown that mycotoxins can induce changes in the expression levels of miRNAs in body cells. Differences do arise, however, depending on the test model, type of toxin, and dose used, as illustrated in Table 1.

**Table 1:** Change in miRNA expression in various subjects caused by the four mycotoxins

Mycotoxins	Dose	Test object	Up	Down	Reference
OTA	200 μg/kg	Piglet	miR-497 miR-133a-3p miR-423-3p miR-34a miR-542-3p	miR-421-3p miR-490 miR-9840-3p	[16]
	1000 nmol/L	NRK-52E NRK-49F	miR-21 miR-200a	\	[17]
	25 μmol/L	LLC-PK-1	miR-200c miR-29c miR-29b miR-132	miR-17 miR-192 miR-200b	[18]
	210 μg/kg	Rats	miR-3596b miR-653 miR-3065-3p	miR-129 miR-130b miR-141	[19]
ZEA	50 μmol/L	TM3	miR-19a-3p miR-96-5p miR-221-5p miR-3057-5p	miR-146-3p miR-3095-5p miR-185-3p miR-467e-3p	[20]
	58 mg/kg	Piglets	miR-1 miR-424-5p miR-452-3p	\	[21]
	40 μg/kg	*Sus scrofa*	miR-15a miR-21 miR-192	\	[22]
AFB1	200 μg/kg	F344 Rats	miR-122-5p miR-34a-5p miR-181c-3p	\	[23]
	1.5 mg/kg	F344 male Rats	miR-146a miR-24 miR-199a-3p miR-23a	miR-122 miR-192 miR-101b miR-30a	[24]
	200 μg/kg	Rats	miR-182 miR-10b-5p miR-224-5p miR-122-5p	miR-802-5p	[25]
	200 μg/kg	Rats	miR-34a-5p miR-200b-3p miR-429	miR-130a-3p	[26]
DON	1.6 μg/mL	JPEC-J2	miR-181a miR-30c miR-365-5p miR-769-3p	\	[27]

The early diagnosis of mycotoxin pathogenicity is generally limited to assays aimed at the protein molecular level, while the detection of miRNAs is often neglected. Because microRNAs exist in biological tissues and body fluids, analyzing and identifying differentially expressed miRNAs can give us valuable additional information that can be used to help diagnose the occurrence of disease. MiRNA has high specificity and sensitivity, so it can be used for reflecting the existence of early-stage diseases, the development of advanced-stage diseases and disease prognosis prediction, and drug resistance [28]. Moreover, interfering with miRNA expression in an appropriate manner can be expected to be a crucial step in inhibiting the occurrence of mycotoxin toxicity.

## Main mechanisms of miRNAs

### Ochratoxin A

At present, in the mechanism of OTA-induced toxicity, OTA mainly activates biotoxicity through oxidative stress, cell apoptosis, and autophagy [29]. Oxidative stress is often a potential factor that induces disease through many adverse reactions, such as DNA damage, protein damage, and lipid damage [30–32]. At the same time, it was found in vivo that after OTA treatment, lipid peroxide (LPO) was significantly increased in the liver, and GSH, SOD, CAT, GR, and GSH-Px were significantly decreased, indicating that the antioxidant capacity of the liver was decreased and toxicity was activated under the influence of OTA [33]. Studies have shown that OTA-induced apoptosis is not only observed in the kidney of mice and rats, but also in vitro cells such as HepG2 cells, HEK293 cells, PK15 cells, and MDCK-C7 cells [34–37]. OTA induces cell apoptosis, mainly through ERK1/2, p38, MAPK, and JNK signaling pathways [29]. Another study has shown that continuous activation of c-Met/PI3K/Akt and MEK/ERK1/2 signaling pathways can be observed in human renal cells after OTA exposure [38]. All this evidence suggests that apoptosis is one of the modes of OTA-induced cytotoxicity. Autophagy is an adaptive response of the body to fight disease, and the process of autophagy is often accompanied by the adaptive process of mitosis to protect the body from the damage of toxic substances [39, 40]. It has been reported that mitochondrial dysfunction tends to occur in the early stage of OTA toxicity, and HEK293 cells lacking the mitotic receptor Nix are more susceptible to OTA toxicity [41]. Thus, Nix plays a key role in autophagy and mitosis, protecting the body from the effects of OTA.

**Fig. 1:**
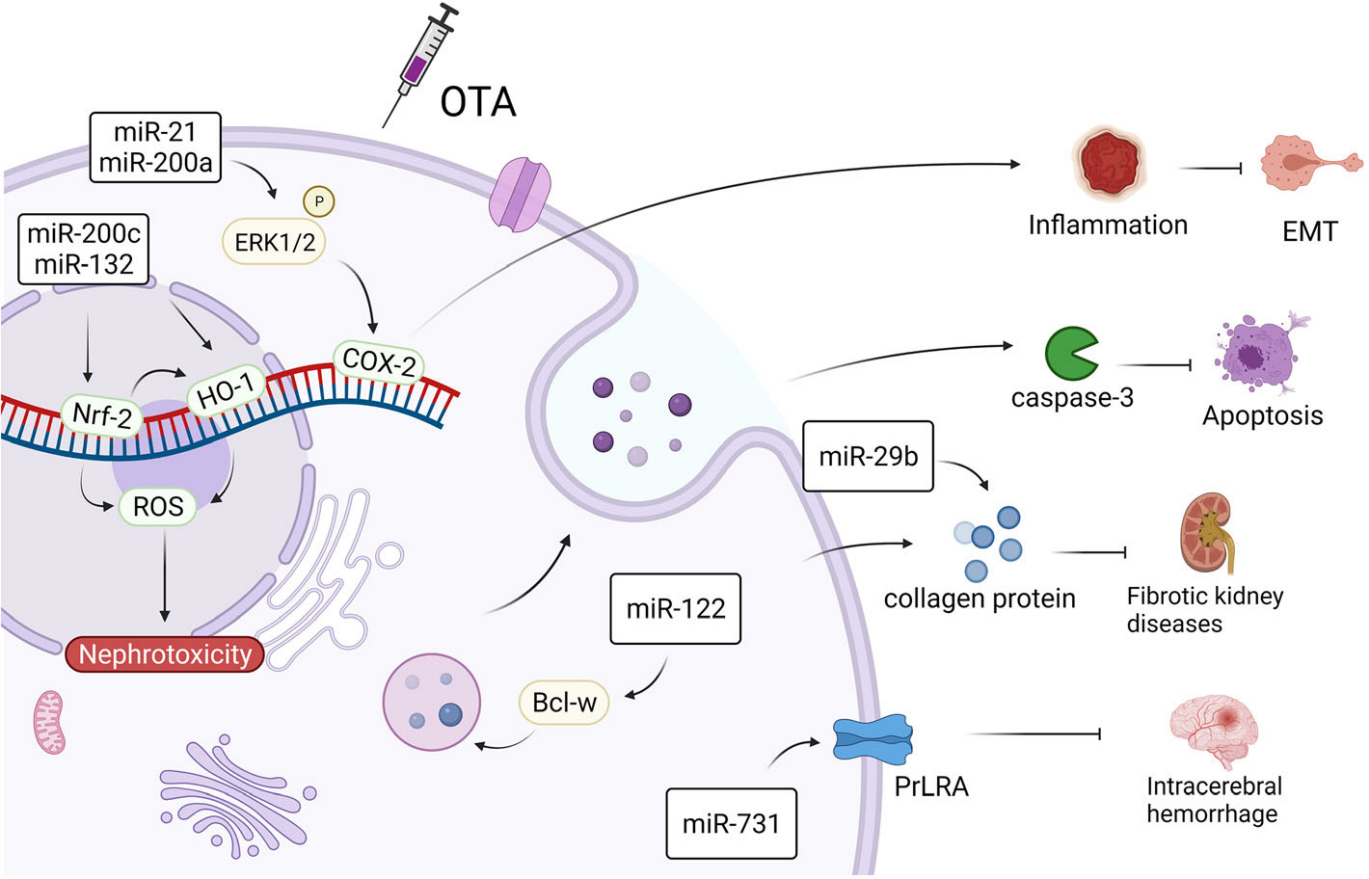
Mechanisms of OTA toxicity regulated by miRNAs. After OTA exposure, by activating miR-21 and miR-200a in cells, it significantly phosphorylates ERK1/2 and induces the activation and expression of COX-2, which in turn induces the occurrence of inflammation and EMT in the body and leads to cytotoxicity. OTA can indirectly inhibit HO-1 expression of inhibition of Nrf2 signaling by miR-132 or activation of miR-200c, thereby increasing ROS levels in vivo and increasing nephrotoxicity. OTA can activate collagen expression of miR-29b and cause renal fibrosis. OTA can inhibit the prolactin receptor (PRLRA) by miR-731 and induce cerebral hemorrhage. OTA activates Bcl-W via miR-122, which in turn stimulates caspase-3 and triggers apoptosis

It has been reported that the target organ of OTA is the kidney, so the nephrotoxicity is particularly prominent [42]. Proximal tubular interstitium damage can also be observed in livestock fed with OTA-containing feed, resulting in serious renal toxicity and even renal tumors in severe cases [43, 44]. Chronic kidney disease (CKD) has a global incidence of 11–13% and kills up to 1.2 million people annually [45]. At this stage, we have also summarized the mechanism of OTA toxicity, which is shown in Fig. 1. For example, NRK-52E (rat renal tubular epithelial cells) and NRK-49F (rat renal fibroblasts) co-culture models treated with OTA showed that miR-21 and miR-200a significantly phosphorylated ERK1/2 and induced activation and expression of COX-2, showing significant inflammatory and fibrotic responses. This is also a key factor in inducing EMT (epithelial-mesenchymal transition) [17]. Some studies have also pointed out that changes in the expression levels of miR-21 and miR-382 can be detected in acute and chronic kidney diseases, and these indicators play a key role in the development of fibrosis [46]. Another hotspot of OTA toxicity studies is the nuclear factor erythroid 2-related factor 2(NRF2)-related signaling pathway. Studies have shown that OTA can inhibit the expression of Nrf2 and HO-1 through miR-132 and miR-200c, and induce OTA toxicity by increasing the level of ROS in vitro [18]. Similar results were also found in the OTA-fed rat model, which showed that miR-141 significantly enhanced the activity of KEAP1, thereby inhibiting the expression of Nrf2, and activating OTA toxicity in rats [19]. In HEK293 cells, OTA exposure significantly reduced the effect of miR-29b and thus enhanced collagen expression, both of which could significantly increase the risk of fibrotic nephrotic disease [47]. It can also be found in zebrafish embryos that OTA acts on miR-731 to inhibit the prolactin receptor (PrLRA) and induce cerebral hemorrhage [48].

To protect itself from OTA toxicity, the body can activate a protective pathway that blocks the cell cycle as the toxicity develops and initiates apoptotic mechanisms. ShRNA-mediated Nrf2 inhibition increased OTA sensitivity, and the P53 signaling pathway significantly activates the expression of miR-34 under the stimulation of OTA, leading to increased expression of *PUMA*, *p21*, and *c-myc*, thus inducing apoptosis [49]. It has also been reported that OTA increases the expression of miR-122 in GC-2 cells, which triggers apoptosis after caspase-3 activation by inhibiting Bcl-w [50].

Studies have shown that histone acetylation is also involved in the regulation of OTA toxicity and carcinogenesis. It is known that histone acetylation and deacetylation are closely related to gene transcription and expression and are influenced by acetylase (HATS) and deacetylase regulation (HDACs) [51]. OTA has been reported to be involved in mitotic stagnation by inhibiting Hats activity in the nucleus and regulating acetylation of histone and non-histone lysine residues in a dose-dependent manner [52]. Increased HDAC activity and atypical PKC phosphorylation were also observed in the kidneys of OTA-fed male F344 rats, which are associated with MAPK extracellular regulation of selective downstream activation of ERK1/2 and its substrate ELK1/2 and p90RSK [53]. At present, PKC and MEK/ERK1/2 and MAPK signaling pathways have been shown to play a key role in cell proliferation, apoptosis, and cancer development [53, 54]. Thus, the histone modification layer plays a potential role in OTA-induced biotoxicity.

### Aflatoxin B1

There are many aflatoxins (AFs) but AFB1 is the most toxic. It is a potent human carcinogen (group 1 classified). Due to its pervasiveness and strong toxicity, AFB1 is often regarded as the main object of interest in mycotoxin research [55, 56]. The molecular structure of AFB1 is based on a derivative of dihydrofuran and coumarin, which is an indirect carcinogen. Liver cancer ranks fourth in the incidence of solid tumors worldwide with a mortality rate ranking third among all cancer subtypes, and Hepatocellular carcinoma (HCC) accounts for 75–85% of primary liver cancers [57].

When the body is exposed to AFB1, the *p53* gene in the body is prone to genetic mutation. At this time, guanine (G) is replaced by thymine (T), which results in the arginine located at site 249 in p53 protein changing into serine [58]. It has been reported that the *p53* gene is a tumor-suppressing gene in cells and plays a key role in regulating the cell cycle, apoptosis, autophagy, and DNA repair [59]. The mutated *p53* gene can be detected in most cancer patients: its ability to inhibit the occurrence of cancer is reduced, leading to the induction of pathological changes [60].

When AFB1 is ingested by the body, 50% of it is absorbed by the duodenum. It binds to the plasma albumin and enters the liver: the unabsorbed part is excreted in stools. The toxicity to the body is not due to the AFB1 itself but the toxic effects of the electrophilic and highly active major carcinogens generated after cytochrome P450 is transformed in the liver (including AFB1–8,9-epoxy compounds). AFB1 may also be metabolized to aflatoxins that are slightly less mutagenic, e.g. M1 (AFM1), Q1 (AFQ1), or P1 (AFP1) [61–63]. When the P450 system in the liver promotes AFB1 metabolism, it produces a large amount of ROS, which can enhance the toxic effect of the AFs. These free radicals can damage cell membranes and soluble cell material. This eventually damages cell function and leads to cell lysis, directly causing cytotoxicity [64]. AFB1 also inhibits protein synthesis, thus interfering with the synthesis of the enzymes required for metabolism, energy, and fat transport processes [65].

HCC occupies third place in the total number of cancer cases worldwide, while HCC accounts for 75–85% of HCC cases with a survival rate of less than 16% [57]. AFB1 is one of the most commonly-encountered mycotoxins and it is believed that dietary exposure to it causes hot mutation of *p53* and significantly increases the probability of HCC developing [66]. It has been reported that the expression levels of *Drosha* and *Dicer* genes decrease significantly after the addition of 10 μg/mL AFB1 to HepG2 cells, thus demonstrating that miRNAs are involved in the biological processes by which AFB1 induces HCC [67]. At the same time, miRNAs can be used as serum identification criteria for HCC and other tumors. For example, the expression levels of miR-122-5p, miR-24, and miR-802-5p in rat serum can be used as indicators for the diagnosis of HCC in its early stages. Similarly, the overexpression of miR-24 and miR-122 in HCC cells can be used as a prognostic factor for HCC [68, 69].

**Fig. 2:**
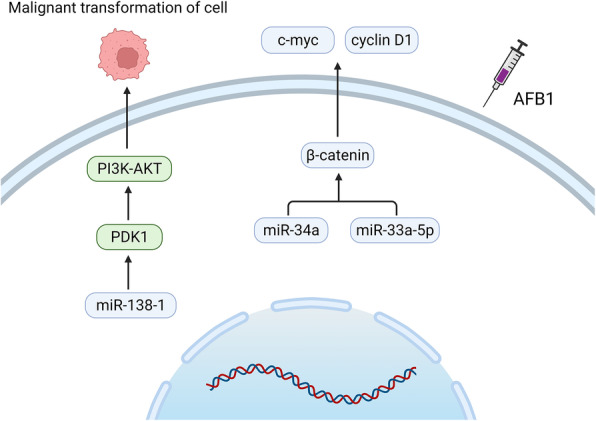
Mechanisms of AFB1 toxicity regulated by miRNAs. After exposure to AFB1, miR-34a and miR-33a-5p inhibit the expression of β-catenin in the Wnt signaling pathway, which in turn causes the decrease of *c-myc* and cyclin D1, and results in the arrest of cell cycle S phase and the risk of HCC. Overexpression of miR-138-1 can significantly inhibit the activation of PDK1, thereby inhibiting the expression of related proteins in the PI3K/Akt signaling pathway, and ultimately alleviating the malignant transformation of cells caused by AFB1

It has been shown that miRNAs play roles in the pathogenesis of various cancers by targeting cancer genes and oncogenes. For example, AFB1 significantly increases the expression of miR-34a in HepG2 cells, leading to a significant decrease in β-catenin, *c-myc*, and cyclin D1 in the Wnt signaling pathway, subsequent arrest of the S phase of the cell cycle, and increase in the risk of HCC [67]. Some studies have indicated that AFB1 can also significantly increase the expression of miR-33a-5p in HepG2 and inhibit the Wnt/β-catenin signaling pathway [70]. Thus, AFB1-induced miRNAs play a key role in the generation of HCC in the Wnt signaling pathway. Similarly, AFB1 is known to increase the incidence of lung cancer. However, targeted regulation of miRNAs can effectively intervene in the occurrence of AFB1-induced lung cancer. For example, it has been reported that the overexpression of miR-138-1 in human bronchial epithelial cells (P50 B-2A13 cells) can inhibit 3-phosphoinositide-dependent protein kinase-1 (PDK1) expression. This inhibits the expression of downstream PI3K/Akt-related proteins which reduces AFB1-induced malignant transformation in the P50 B-2A13 cells, and significantly reduces the incidence of lung cancer in the body (Fig. 2) [71].

MiRNAs also play important roles in the apoptosis of tumor cells. It has been reported that miR-429 is significantly up-regulated in HCC tumor tissues, promoting their proliferation and inhibiting their apoptosis [72]. Furthermore, the expression level of miR-429 was found to be correlated with the size of the tumor. It has also been noted that miRNA-429 has a significant oncogenic effect, promoting the occurrence of pancreatic ductal carcinoma and gastric and rectal cancer by targeting the expression of EP-300, SOX2, and *c-myc* [73, 74]. Inhibiting the expression of miR-429, on the other hand, can significantly inhibit the proliferation of tumor cells and promote their apoptosis [72].

AFB1 can also exert biotoxicity via epigenetic modification [75]. It has been reported that the levels of transcriptional activation markers H3K9me3 and H4K20me3 are raised in oocytes of mice fed AFB1, while the level of transcriptional inhibition marker H3K27me3 is lowered [76]. These results are associated with increased levels of DNA methylation in the genome which significantly inhibits reproductive function in mice. Similar results have been observed in porcine oocytes, where the levels of transcriptional activation markers H3K27me3 and H3K4me2 are reduced, while the level of transcriptional inhibition marker H3K9me3 is increased [77].

### Deoxynivalenol

One of the reasons that DON is toxic is that it directly activates ribosome-associated kinases, e.g. the double-stranded RNA associated protein kinase R (PKR), which activates eukaryotic initiation factor 2α (eIF2α) and inhibits protein transcription and translation [78]. PKR can be used as a ligand-activated protein kinase pathway, such as the p38/JNK and ERK signaling pathways, to further promote cell proliferation, differentiation, and apoptosis, and activate the ‘ribosomal stress response’ to cause cytotoxicity [79].

Another pathway by which DON induces toxicity in the body is through the mitochondrial stress response. DON can disrupt the redox system, inducing an imbalance in the redox homeostasis in the body and causing damage to lipids, proteins, and DNA [80]. Cell apoptosis can be induced by promoting the release of mitochondrial cytochrome C and activation of members of the caspase family. DON reduces the transmembrane ability of mitochondria, releasing overloaded peroxide ions to make the ions on both sides of the mitochondrial membrane unbalanced. Rupture of the mitochondrial outer membrane leads to a change in the membrane permeability transition pore (PTP), finally promoting cell apoptosis and causing cytotoxicity [81, 82].

As a regulator of intracellular steroid production, DON can use granulocytes to regulate the secretion of progesterone, testosterone, and estradiol in vivo, promoting the development of oocytes and embryos and participating in reproductive development [83]. At the same time, DON may also be involved in steroid production by regulating miRNAs. For example, miR-181a, miR-23a, and miR-26b, which are seriously affected by DON, regulate the expression of progesterone receptors and endanger that normal reproductive function is maintained in animals [84].

**Fig. 3:**
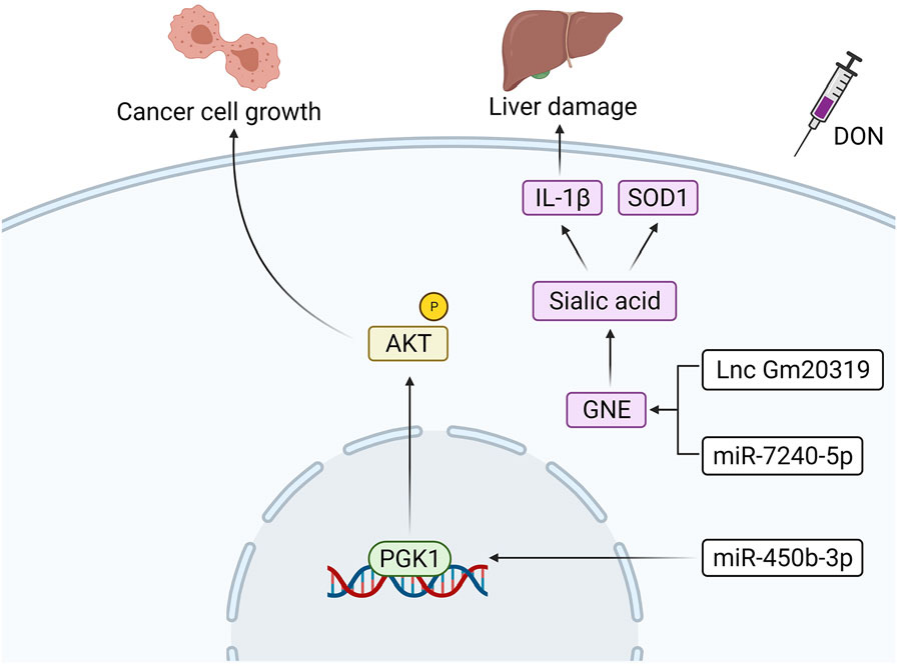
Mechanisms of DON toxicity regulated by miRNAs. The low expression of miR-450b-3p in HCC cells inhibits the expression of its target gene *PGK1*, and then promotes the proliferation of cancer cells by inhibiting the phosphorylation of Akt. After exposure to DON, the combination of lncRNA GM20319 and miR-7240-5p significantly inhibits the expression of *GNE* gene, which in turn stimulates the decrease of sialic acid activity, the decrease of SOD1 expression, and the upregulation of IL-1β expression, and finally triggers liver injury

It is now known that DON is involved in the activation of MAPKs (including p38, c-Jun N-terminal kinase, and ERK1/2) by binding to 60S ribosomal subunits and inhibiting protein transcription and translation [85, 86]. At the same time, miRNAs may also influence the activation of MAPK signals and so the expression of miRNAs plays an important role in the mechanisms responsible for DON’s toxicity. For example, in female pigs, DON can cause significant upregulation of miR-21 and activate the ERK-MAPK pathway, inducing biotoxicity. Compared with normal liver cells, the expression level of miR-450b-3p in HCC cells is significantly reduced, the mRNA expression level of its target gene *PGK1* is inhibited, and the phosphorylation of Akt also significantly inhibited. This promotes the proliferation of HCC cells, inducing the formation of HCC tumors (Fig. 3) [87]. When C57BL/6 mice were treated with 25 μg/kg DON, it was found that lncRNA GM20319 was able also bind with miR-7240-5p to significantly down-regulate the expression of *GNE* genes, inhibiting the activity of sialic acid, changing the expression of IL-1β and SOD1 in the mice’s livers, and triggering liver injury (Fig. 3) [88].

It has been reported that miRNAs present in exosomes also play a key role in DON-induced biotoxicity. Exosomes measuring 40–100 nm are widely present in the emulsion. They can encapsulate proteins, mRNA, miRNAs, deoxyribonucleotides, and lipids, thus preventing their degradation and allowing them to perform biological activities by transporting them to specific cells [89]. As a result, exosomes can effectively prevent the DON-induced destruction of tight junction proteins in intestinal cells, promote the development of neonatal intestinal tract, and inhibit cell apoptosis. They can also enter immune cells through endocytosis and play an important role in immune function [90, 91]. The overexpression of miR-181a, miR-30c, miR-365-5p, and miR-768-3p in porcine milk exosomes can significantly down-regulate the expression of their target genes in the P53 signaling pathway. P-Akt and β-catenin are up-regulated to reduce the damage caused by DON [92]. Meanwhile, it has been found that miR-125b in milk exosomes can target P53 inactivation [92]. Other research has pointed out that several other miRNAs, including miR-30d, miR-25, and miR-504, can also weaken the transduction of the P53 signal and reduce the toxicity of DON [93].

DON can significantly reduce the expression of p-MAPK in porcine oocytes, disrupting spindle formation and inducing cell arrest and inhibiting oocyte development. At the same time, DON causes increased DNA methylation in porcine oocytes by changing the expression level of *DNMT3A* mRNA. Elevated protein expression of H3K27me3 and H3K4me2 and mRNA expression of related methyltransferase genes *SUV39H2*, *SETDB1*, and *EZH2* result in increased histone methylation [94]. In conclusion, DON can affect the maturation of porcine oocytes through epigenetic modification [94, 95].

### Zearalenone

The toxicity of ZEA arises from its role as an endocrine disruptor, which leads to estrogen and pituitary hormone disorder and damages gonadal function. Its structure is similar to that of 17β-estradiol, allowing it to competitively bind to estrogen receptors (ERs). ZEA binds to ERs to form homologs or heterodimers and binds to estrogen receptor elements in the nucleus so that it participates in the transcription of estrogen-responsive genes [96, 97]. The reproductive toxicity of ZEA to livestock is particularly obvious: decreased ovulation, increased rates of stillbirth and dystocia, altered reproductive tracts, and decreased reproductive function [98]. It has also been reported that the Kisspeptin-GPR54-GnRH signaling pathway in the hypothalamus is activated in adolescent rats after ZEA ingestion. Uterus enlargement and vaginal opening have also been observed in adolescent rats, suggesting that ZEA can cause toxicity by affecting pituitary function [99]. ZEA also severely affects the activity of testes and ovaries in mice, pigs, and cattle [100–102]. For example, ZEA has been found to significantly enhance apoptosis of mouse testicular mesenchymal cells and oocytes, interfere with oocyte maturation and development, and arrest cell cycle [103]. Significant increases in the weights of ovaries and uteri and number of follicles were also observed [104]. These results indicate that the endocrine-disrupting function of ZEA is closely related to damage caused to the gonads.

It is well known that ZEA is an exogenous endocrine-disrupting substance that mainly exerts biotoxicity by affecting the reproductive systems of livestock and poultry [105]. Pigs are one of the most sensitive species to ZEA, exposure leading to decreased ovulation, reproductive disorder, reduced birth rate, and fetal abnormalities [106]. It has also been reported that the signal transduction of estrogen plays a role through G protein-coupled receptors, and the expression of miRNAs in vivo is changed after being affected by estrogen, thus affecting estrogen receptor α (ERα), which is closely related to the damage mechanism of ZEA [107, 108]. In vivo experiments in pigs have shown that ZEA activates the PKC and p38 signaling pathways through GRP30 (a G protein-coupled receptor) on the cell membrane. At this time, the activated miR-7 targets the activation of the *FOS* gene and identifies the synthesis and secretion of FSH, seriously affecting the reproductive health of female animals [109]. Estradiol, the most active form of natural estrogen, can significantly enhance the proliferation and migration of human ovarian cancer cells (PEO1) through ERα. Also, it has been found that the expression levels of miR-200, miR-203, and miR-203a in PEO1 cells are significantly dependent on ERα, inhibiting the expression of E-cadherin, promoting the expression of ZEB1 (Zinc Finger E-box-Binding Homeobox 1), and inducing the formation of EMT [110].

**Fig. 4:**
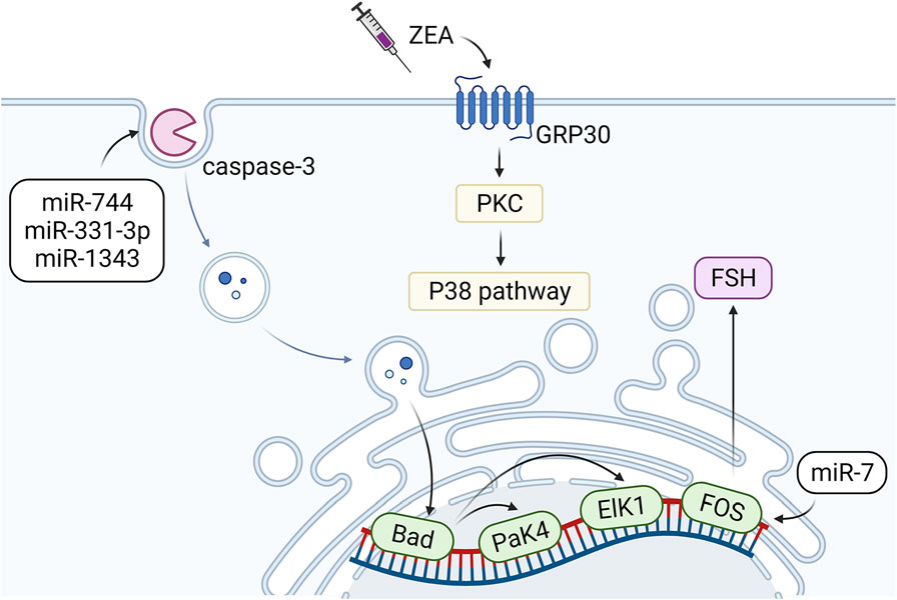
Mechanisms of ZEA toxicity regulated by miRNAs. After ZEA is exposed to the cell membrane, it activates PKC through the membrane surface protein GRP30, and induces cytotoxicity by activating the P38 signaling pathway. Overexpressed miR-7 can recognize the synthesis and secretion of FSH through targeted regulation of* FOS* gene expression, which seriously affects the normal estrogen secretion of female animals and induces ZEA toxicity. ZEA triggers apoptosis and cytotoxicity by activating the apoptotic executioner caspase3, which is specifically manifested as the overexpression of intracellular miR-1343, miR-331-3p, and miR-744, the expression level of *PaK4* and *ElK1* decreases, and the expression of *Bad* rise

ZEA can also affect reproduction through an apoptotic pathway regulated by miRNAs [109]. It has been reported that 30 μmol/L of ZEA in vivo can enhance the apoptosis gene *Bad* and activate caspase by activating miR-1343, miR-331-3p, and miR-744 which down-regulates the expression levels of apoptosis-related genes *PAK4* and *ElK1* (Fig. 4) [111]. *PAK4* and *ElK1* have a variety of cellular functions and play a role in cell growth, movement, apoptosis, and other processes [112]. In conclusion, miRNA plays a key role in regulating reproductive injury caused by ZEA and plays a key role in animal production and medical treatment.

Epigenetic modification is an important regulatory factor in spermatogenesis. For example, DNA methylation and histone modification play crucial roles in spermatogenesis and development [113, 114]. It has been reported that sperm are susceptible to epigenetic interference, and even show a cross-generational epigenetic marker which can lead to male infertility, embryo development failure, and disease in offspring, etc. [115]. Studies have found significantly increased activity of G9a and H3K9Me2 in the testes of male mice after exposure to ZEA [116]. G9a is known to be an important histone methyltransferase that is involved in the monomethylation and dimethylation of H3K9 (H3K9Me1 and H3K9Me2) [117]. It has also been pointed out that G9a and H3K9 are also involved in sperm meiosis [118]. In conclusion, G9a and H3K9 are closely related to ZEA-induced sperm impairment.

## Conclusions and prospects

This review has mainly concentrated on the expression status of miRNAs in the toxicological processes induced by OTA, AFB1, DON, and ZEA, including their target genes and their molecular mechanisms of action in their signaling pathways. This study further confirms the importance of miRNAs in the pathogenesis of mycotoxins and their roles as biomarkers in the prevention and regulation of toxins. Further understanding the function of mycotoxin-related miRNA, the interaction between miRNA and target, and the relationship between miRNA-mediated gene expression are very important to explain the toxicodynamics of mycotoxin. The changes in miRNA levels provide new insights into the mechanism of mycotoxin-induced toxicity and can be used as a marker for disease diagnosis and prognosis.

This paper points out that many pathways related to the action mechanism of mycotoxins, such as ERK1/2, Nrf2, p38, MAPK, and Akt, are involved in the regulation mechanism of miRNA. In addition, this paper also summarizes the mechanism of histone modification between mycotoxins. Although the expression levels of miRNAs have been clearly elucidated in the relevant articles, our knowledge of their specific functions and mechanisms of action is still imperfect. Therefore, there is an urgent need to conduct further research on miRNA pathways and use the information gained to overcome the toxic effects of these (and other) mycotoxins.

## Data Availability

All data generated or analysed during this study are included in this published article.

## Abbreviation

OTA: Ochratoxin A
AFB1: Aflatoxin B1
DON: Deoxynivalenol
ZEA: Zearalenone
miRNAs: MicroRNAs
NRF2: Nuclearrespiratoty factor 2
PI3K: Phosphoinositide 3-kinase
GSH: Glutathione
SOD: Superoxide dismutase
CAT: Catalase
GR: Glutathione reductase
GSH-Px: Glutathione peroxidase
HDAC: Histone deacetylase
PKC: Protein kinase C
CKD: Chronic kidney disease
EMT: Epithelial-mesenchymal transformation
COX-2: Cyclooxygenase-2
PrLRA: Prolactin receptor
AFs: Aflatoxins
HCC: Hepatocellular carcinoma
HepG2 cells: Human hepatoma cells
P50 B-2A13 cells: Human bronchial epithelial cells
PDK1: 3-phosphoinositide-dependent protein kinase-1
EP-300: E1A binding protein p300
PKR: Protein kinase R
eIF2α: Eukaryotic initiation factor 2α
PTP: Permeability transition pore
ERs: Estrogen receptors
GRP30: G protein-coupled receptor
PEO1: Human ovarian cancer cells
ZEB1: Zinc Finger E-box-Binding Homeobox 1
MAPK: Mitogen-activated protein kinases
ERα: Estrogen receptor α
